# Closing the Gaps: From Science to Action in Maternal, Newborn, and Child Health in Africa

**DOI:** 10.1371/journal.pmed.1000298

**Published:** 2010-06-29

**Authors:** Sara Bennett, Freddie Ssengooba

**Affiliations:** 1 Health Systems Program, Johns Hopkins School of Public Health, Baltimore, Maryland, United States of America; 2 School of Public Health, Makerere University, Kampala, Uganda

## Abstract

As part of a series on maternal, neonatal, and child health in sub-Saharan Africa, Sara Bennett and Freddie Ssengooba discuss the challenges of getting science into policy in Africa.

This paper is part of a *PLoS Medicine* series on maternal, neonatal, and child health in Africa.

The previous papers in the *PLoS Medicine* series [1],[2] demonstrate that the technical basis for improving maternal, newborn, and child health (MNCH) in sub-Saharan Africa is largely known, but too often policy and practice are not well informed by science. There are two distinct aspects to this “gap.” First there is a “science to policy and practice” gap. Accumulated scientific research on the severity of MNCH problems and strategies to promote MNCH has, at least in part, failed to ensure that MNCH reaches the domestic policy agendas of African countries, and stays there. Furthermore, local, context-specific evidence frequently is not applied in planning and programming interventions to address MNCH. Second there is a “policy to practice” gap: even where clear policy commitments to MNCH are made, there may be substantial challenges to getting such policies implemented. These include challenges related to stakeholder management through the implementation process and challenges associated with the negotiation of health system constraints. Many African countries face weakened health systems characterized by human resource shortages, dysfunctional drug supply systems, decaying health infrastructure, and weak supervisory and governance mechanisms. Consequently, the global community is currently strongly focused on strengthening health systems [3] so that they can provide adequate platforms for the delivery of a range of services, including MNCH.

Our discussion focuses on the “science to policy and practice” gap, in the belief that action to address the second gap is already mobilized, although clearly not yet fully effective. In contrast, the first gap remains neglected. This article first addresses what is already known about how scientific evidence has influenced MNCH policy and practice, then it considers some of the key challenges in closing the science to policy and practice gap, and concludes by identifying promising paths for future action.

Global and country-specific evidence on maternal and child mortality, service coverage, and effective interventions to improve MNCH has been key to stimulating greater global attention to these issues through monitoring efforts such as those of the Countdown group, which tracks progress towards the Millennium Development Goals (MDGs) that address MNCH. However, much more needs to be done to ensure that MNCH issues reach national policy agendas and that they remain a high priority given the importance of policy consistency in promoting the MNCH agenda [1]. The Countdown project assessed national policy for MNCH through select policy indicators (for example, the adoption and enactment of the International code on Marketing of Breast Milk Substitutes and the presence of a costed implementation plan for MNCH) [4]. It concluded that, while policies had improved in the 68 priority countries, policy environments were not yet fully supportive and a major gap remains between policy and action.

Such indicators, however, are probably relatively insensitive measures of the true political priority given by African leaders to MNCH. Shiffman [5] analyzed the political priority given to maternal mortality. Through national-level interviews and document review he assessed the extent to which: (1) national political leaders expressed sustained concern for the issue; (2) the government enacted policies that embraced strategies to address the problem; and (3) the government allocated and released public budgets commensurate with the problem's gravity. He rated the political priority accorded to maternal mortality as low in the one African country included in the study. Despite intensive global advocacy efforts, MNCH may not be a high policy priority for many African governments.

Where there is commitment to MNCH and an intention to support action to address MNCH issues, African countries need to tailor strategies to match health system capacity. Local data need to be compiled and analyzed to guide how MNCH service packages can be integrated and delivered within the given resource constraints. Evidence as to the use of MNCH data in health planning is limited, but we know from multiple sources that, in general, data quality is poor and the use of data for planning and decision making is weak [6].

## Challenges to Closing the MNCH Science to Policy and Practice Gaps

While the MDGs, including MDGs 4 and 5, remain the cornerstone of government and development partner policies, the harsh reality is that African Ministries of Health face multiple competing priorities. Obviously HIV/AIDS and other infectious diseases continue to demand time and attention, but so do health worker concerns about pay, issues of drug shortages, and emerging concerns about the health effects of climate change and chronic disease. In such contexts it is naïve to think that Ministries or Ministers can remain consistently focused on critical MNCH issues. Broader policy and advocacy coalitions need to be developed and employed to help promote government accountability and maintain focus.

Unlike HIV/AIDS, for which a discrete and close-knit group of affected persons may be organized for effective advocacy, MNCH beneficiaries are often diffuse and not organized into a strong lobby. While the White Ribbon Alliance, an international coalition that advocates and raises awareness concerning safe motherhood, has had substantial achievements in raising the profile of maternal health issues, advocacy coalitions for MNCH in Sub-Saharan African countries are few. For example, this Alliance has branches in only nine sub-Saharan African countries. Furthermore, MNCH does not lend itself to a simple, “silver-bullet” fix. Instead it raises a set of more complex (yet still tractable) and context-specific policy issues: are formal health services financially and geographically accessible to women? Do policies support community-based workers who can identify and refer high-risk women? Are there policies and programs in place that promote appropriate nutrition for girls and women throughout their lives? Such complex policy questions need to be complemented by implementation research that supports policy adaptation to local contexts.

Certain projects have demonstrated that considerable health impacts can be achieved through the local interpretation and application of data to MNCH planning and programming. For example, in Nepal an intervention that enabled women's groups to review local evidence, and to jointly plan, implement, and assess interventions aimed at addressing local perinatal problems led to increased coverage of antenatal care and attended deliveries, and ultimately to a substantial drop in neonatal mortality [7]. However such projects have typically operated on a relatively small scale and with quite intensive technical support. In many contexts the quality of routine data is poor, and health staff and community capacity to analyze data are limited. Even when these two primary obstacles have been tackled, the lack of an organizational culture that supports evidence-informed decision making has remained problematic [8]. Given the decentralized nature of many African health systems, analytical skills and a culture of evidence-informed decision-making need to be developed in district management teams and front-line health workers, as well as in Ministries of Health. Staff at all of these levels need to be empowered to generate and use data and operations research findings in their planning and decision making processes.

## What Must Be Done

The challenges in bridging the science to policy and practice gap are considerable, but they are not insurmountable. As for MNCH service packages, interventions to strengthen the use of science in policy and decision making will have positive ramifications for the whole health system, enabling the impact of ongoing health systems strengthening investments to be multiplied. Not all of the challenges identified above can be addressed: we propose three strategies that we believe would have the greatest impact on MNCH.

### 1. Develop MNCH Policy Networks

One of the key developments in policy during the past decade has been an increasing understanding of the importance of the “webs of influence” that guide the exercise of power. Securing and sustaining national political priority for MNCH must go beyond politicians and ministers and engage civil society, front-line health workers, researchers, and the media. This cannot be achieved in a “top-down fashion,” instead it should be stimulated by small pots of funds to foster the emergence of grassroots groups and coalitions. Powerful and persuasive evidence that can be generated through the Lives Saved Tool (LiST) [9], a computer-based tool that allows users to predict the impact of alternative packages of MNCH services, needs to be packaged and communicated in ways that are easily accessible to all of these different groups. Such policy networks can reinforce chains of mutual accountability, particularly when evidence regarding progress against MNCH goals is available.

### 2. Mainstream the Use of MNCH Science

Given the multiple competing demands on decision makers it is critical to ensure that the analysis and application of evidence to support MNCH fit into planning and monitoring processes, again strengthening the overall process of health planning rather than adding an extra burden. For example, rigorous efforts to understand trends in MNCH and to plan services to address MNCH should be integrated into poverty reduction strategies, sector-wide approaches, and other ongoing processes that require the use of evidence in policy and resource allocation decisions.

### 3. Invest in Innovative Approaches to Develop and Apply MNCH Evidence

While there have been substantial international efforts aimed at synthesizing and packaging evidence so as to influence MNCH policy, by and large this has not been replicated at national levels. The ability to generate, synthesize, and apply evidence of different sorts—health data, global health research, and experiences of health practitioners and communities—is critical to the development of adaptive health systems able continually to strengthen MNCH services. While some investment has been made in developing-country research capacities [10], much more is needed to build local institutions that can conduct relevant research for MNCH policy and implementation. Equally critical is investment in enhancing skills and capacities to apply such research findings in practice and in programs. This is needed not only among policy makers but across the whole range of actors involved in MNCH policy networks.

As the main papers in the PLoS Medicine series suggest [1],[2], there are a growing number of answers about what is clinically and programmatically effective in promoting MNCH. While weak health systems continue to be a barrier to the more rapid scale-up of MNCH and other priority services, there has recently been increased attention to health system strengthening. We have argued that the key challenge now is stimulating an approach to MNCH policy development, planning, and management that relies on evidence in its multiple forms, to steer strategy, and to facilitate the tailoring of global solutions to local conditions. Fortunately, there is also a growing body of knowledge about how to promote the appropriate use of science in policy and practice: it is time to begin to apply what we know about effective science to policy and practice strategies to the MNCH field. As we apply what we already know, we should continue to build institutional and individual capacity for the local adaptation and indigenization of global MNCH evidence to national and subnational contexts, resources, and constraints.

## Abbreviations

MDG: Millennium Development Goal
MNCH: maternal, newborn, and child health
